# Evaluation of Erythrocytes, Platelets, and Serum Iron Profile in Dogs with Chronic Enteropathy

**DOI:** 10.4061/2010/716040

**Published:** 2010-07-28

**Authors:** Veronica Marchetti, George Lubas, Andrea Lombardo, Michele Corazza, Grazia Guidi, Giovanni Cardini

**Affiliations:** Department of Veterinary Clinics, University of Pisa, via Livornese lato monte, I-56122 San Piero a Grado, Pisa, Italy

## Abstract

The aim of this study is to evaluate iron status, erythrocyte, and platelet modifications in dogs with chronic enteropathy (CE). Dogs were grouped as food-responsive diarrhea (FRD, *n* = 11), antibiotic-responsive diarrhea (ARD, *n* = 5), and steroid-responsive diarrhea (SRD, *n* = 6) relating to therapeutic-response. Clinical and haematological findings, evidence of gastrointestinal blood loss, and iron metabolism were evaluated before and after treatment. A mild normocytic or microcytic anemia and thrombocytosis were identified, respectively in 18.0% and 31.8% of CE dogs. No significant differences between pre- and posttreatment of hematocrit, haemoglobin, and mean corpuscular volume, platelet count and mean platelet volume were found. Statistical analysis pointed out significant differences between pre- and posttreatment in serum iron (*P* < .03) and unsaturated iron binding capacity (UIBC) (*P* < .01). No significant correlations were found between these parameters and canine Inflammatory Bowel Disease activity index and pattern of CE as well.

## 1. Introduction

Different hematologic abnormalities are often observed in human patients with inflammatory bowel disease (IBD). Anemia is a frequent finding in ulcerative colitis (UC) and Crohn's disease (CD), with reported rates varying between 30% and 50%, respectively. Iron deficiency is the most common cause of anemia in IBD, particularly due to occult chronic gastrointestinal (GI) blood loss. The increased epithelial sloughing in chronic GI inflammation may increase the iron loss. Anemia of chronic disease (ACD) is frequently related to iron deficiency anemia (IDA) in IBD patients [1, 2]. The ACD is a consequence of cytokines activation (i.e., tumor necrosis factor alpha [TNF-*α*], interleukin-1 [IL-1], interferon [IFN]) and synthesis of acute phase proteins [1, 2]. Reduced half-life of erythrocytes, decreased erythropoietin synthesis, and reduced bioavailability of iron are the pathophysiological mechanisms involved in ACD [3]. 

Immune thrombocytopenic purpura is rarely reported in human patients with IBD. The same immunological events that lead to intestinal inflammation are hypothesized to cause also platelets destruction and both abnormalities resolve with treatment [4]. Moreover, thrombocytosis may occur in IBD and probably represents a nonspecific response to the inflammation. An elevated platelet count in human medicine is a well-known recognized marker of IBD activity as thrombopoietin levels are significantly elevated [5, 6]. In addition, iron deficiency is a cause of reactive thrombocytosis, although further investigations will be necessary to elucidate the pathogenetic mechanism [7].

Few reports are available regarding the haematological aspects in canine chronic enteropathy (CE). Ridgway and others [8] reported thrombocytopenia, often with macroplatelets, in seven dogs with IBD; thrombocytopenia appears to be subclinical and in some cases resolved with therapy. No correlation between histopathological severity of inflammation and degree of thrombocytopenia was found. Ristic and Stidworthy [9] described a severe microcytic anemia with moderate regeneration and low serum iron concentration, in two dogs with chronic blood loss associated to CE. Craven and others [10], in a retrospective study on 80 cases of canine IBD, reported the incidence of some haematological abnormalities, such as anemia (12%), thrombocytosis (7%), and thrombocytopenia (13%). The authors hypothesized an association of these abnormalities and IBD. 

Furthermore, to the authors' best knowledge, investigations about serum iron profile in dogs with CE were not performed. The purpose of this prospective study was to evaluate erythrocyte, platelet, and iron abnormalities observed in dogs with CE. 

## 2. Materials and Methods

### 2.1. Clinical Cases

Over a 12-month period, 22 dogs (8 females and 14 males) with clinical signs of CE were included in the prospective treatment trial. Selection criteria for cases included a history of chronic diarrhea with or without vomiting of at least 4 weeks duration (mean 10.3 ± 6 weeks), histopathologic evidence of intestinal inflammatory cellular infiltration, and absence of underlying extraintestinal disorders. Laboratory data performed to rule out underlying disorders included complete blood count (CBC), serum biochemical profile, urinalysis and fecal samples analysis for common parasites and *Giardia spp*. (SNAP Giardia, Idexx Laboratoires, Milan, Italy). Additional tests included assessment of trypsin-like immunoreactivity, cobalamin, and folate serum concentrations, and abdominal bi-dimensional ultrasound examination. The dogs' age ranged from 1.4 to 12 years old (mean 4.8 ± 3.4 years, median 3.5 years) and several breeds and mixed breeds of dogs were represented.

All dogs were classified according to the canine IBD activity index (CIBDAI) scoring system, established by Jergens and others [11], which is based on six gastrointestinal signs: general attitude and activity, appetite, vomiting, stool consistency, stool frequency, and weight loss. The total composite score is determined to be clinically insignificant (score 0–3), mild (score 4-5), moderate (score 6–8), or severe (score 9 or greater).

A sequential trial therapy approach with an elimination diet, antibiotics, and immunosuppressive treatment as a last resort, according to other authors [12] was performed. Initially, all dogs were treated with an elimination diet (Purina HA, Nestlé Purina, Udine, Italy) for 10 days. Dogs responding to the elimination diet in the first 10 days (clinical signs improved or resolved) were assigned to the food-responsive diarrhea (FRD) group. Dogs that did not respond in the first 10 days of treatment (clinical signs persisted while on the elimination diet), an antibiotic treatment with tylosin (20 mg/kg Body Weight [BW] q12h per os [PO] for 4 weeks) was administered. If a positive response to both treatments improves or resolves the symptoms, dogs were assigned to the antibiotic-responsive diarrhea (ARD) group. In dogs that did not respond to antibiotic therapy, oral prednisolone without any antibiotic support was given (1 mg/kg BW q12h PO for 20 days followed by a tapering dosage over 6–8 weeks) and these patients were assigned to the steroid-responsive diarrhea (SRD) group. All dogs were fed continuously with elimination diet for 12 weeks.

At the time when diagnosis was postulated and when clinical signs were improved or resolved, a CBC (HecoVet, Florence, Italy) with reticulocyte count (LaserCyte Idexx Laboratories, Milan, Italy and manual count with new methylene blue; the results obtained were overlapping each other) was performed. The serum iron profile including iron, ferritin, unsaturated iron binding capacity (UIBC), related calculation of total iron binding capacity (TIBC), and transferrin saturation percentage, together with the measurement of C-reactive protein concentration were performed with Olympus AU2700 chemistry-immuno system and using the supplied reagents (Olympus Italia srl, Segrate, Milan, Italy). In addition, the fecal occult blood test (HemoFec, Roche Diagnostics GmbH, Mannheim, Germany) was also carried out. All dogs tested for fecal occult blood underwent to three days meat restriction. Moreover, each clinical sign recorded and body weight were evaluated and scored.

### 2.2. Statistical Analysis

Normally distributed data are reported as mean ± standard deviation and paired Student's *t-*test was used to analyze the differences between data at diagnosis and after treatment. A one-way analysis of variance (ANOVA) and Tukey test were used to evaluate differences among groups of dogs (FRD, ARD, and SRD). Correlations were evaluated with the Spearman correlation test. Statistical significance was set at *P* <  .05. 

## 3. Results

### 3.1. Clinical Findings

Eleven/22 dogs (50%) were assigned to FRD group, 5/22 dogs (22.7%) to ARD group, and 6/22 dogs (27.3%) to SRD group. Six dogs had a clinically not significant disease, 7 had mild, 7 moderate, and 2 severe clinical disease according to the activity index of Jergens and others (2003). The CIBDAI was significantly higher (*P* <  .05) before treatment in SRD group (7.17 ± 2.8) compared with the FRD (5.4 ± 3.2) and ARD (4.6 ± 2.07) groups. After treatment, symptoms were completely resolved in 10 dogs (5/7, 2/5, and 3/6 dogs, respectively in FRD, ARD, and SRD group). The average value of CIBDAI in dogs before treatment was 5.72 ± 2.91 and at the end of therapy (ranging time from 1 month to 6 months) was 0.86 ± 0.99. The reduction in average activity index was statistically significant (*P* <  .001). 

### 3.2. Erythrocyte Findings

At the time of diagnosis and before treatment, a mild normocytic or microcytic anemia was identified in 4/22 dogs (18% of cases; hematocrit, Hct 34.9 ± 2.2%, reference range 37.0–55.0%; mean corpuscular volume, MCV 61.7 +/− 3.3 fL, reference range 60.0–76.0 fL), while microcytosis without anemia was present in 6/22 dogs (27.2%; MCV 58.2 ± 0.8 fL); in 2 of these dogs, positive fecal occult blood test was found. Common findings were anisocytosis (59.0%), polychromasia (40.9%), and Howell-Jolly bodies (13.6%) that were present also at posttreatment evaluation (54.5%, 27.3%, and 4.5%, resp.). At posttreatment time, anemia was identified in 2/22 (9%; mean Hct 35.0 ± 0.1%) of dogs, and one dog with microcytosis was still occurring. No significant differences between pre- and posttreatment in Hct. hemoglobin, MCV, and reticulocytes were found (Table 1). These parameters were not related with CIBDAI and no statistically significant differences were found between FRD, ARD, and SRD groups. 

### 3.3. Fecal Blood Test

The fecal occult blood test was positive in 4/22 of dogs. In one of these dogs, reticulocytosis (72.2 × 10^9^/L, reference range, <60.0 × 10^9^/L) and erythrocytosis (9.1 × 10^12^/L; reference range, 5.0–7.9 × 10^12^/L) were associated. In addition, another dog had erythrocytosis (8.1 × 10^12^/L) and one reticulocytosis (91.9 × 10^9^/L). None of these four dogs showed anemia. 

### 3.4. Serum Iron Profile

Only in one dog with FRD negative for fecal blood test, low serum iron (45 *μ*g/dL, reference range 81–220 *μ*g/dL), low transferrin saturation percentage (17.5%, reference range 25–52%), and concurrent mild normocytic anemia (Hct 32%, MCV 62 fL) were found. Iron deficiency resolved with symptoms (posttreatment serum iron 184 *μ*g/dL, transferrin saturation percentage 49.3%).

Serum ferritin was increased in 2/22 (9%) dogs (226 ± 1.41 ng/mL, reference range 60–190 ng/mL) and TIBC was low in 4/22 (18%) dogs (222.7 ± 28.7 *μ*g/dL; reference range 270–460 *μ*g/dL). Statistical analysis identified significant differences between pre- and posttreatment in serum iron and UIBC as well as TIBC, although transferrin saturation and ferritin did not show any significant differences between pre- and posttreatment evaluations (Table 2). No significant correlation was found between these parameters and CIBDAI or CE pattern.

### 3.5. Serum CRP Concentration

Serum CRP was increased in 15 of 22 dogs (68.2%) before treatment (0.42 ± 0.38 mg/dL; reference range 0–0.15 mg/dL) and in 8 (35.4%) after treatment (0.59 ± 0.51 mg/dL). Serum CRP concentration showed no significant differences between pre- and posttreatment evaluations (Table 2) and did not correlate with CIBDAI and CE pattern.

### 3.6. Platelet Findings

Seven of 22 (31.8%) dogs had thrombocytosis at presentation (510.4 ± 127.9 × 10^9^/L; reference range, 150–400 × 10^9^/L). Thrombocytopenia was not observed in any case. At posttreatment evaluation, thrombocytosis was identified in five (22.7%) dogs (mean PLT 476.8 ± 63.8 × 10^9^/L). No significant differences between pre- and posttreatment platelet count and mean platelet volume (MPV) were found (Table 1). No significant differences were found in serum iron concentration between dogs with thrombocytosis (133.3 ± 33.7 *μ*g/dL, reference range 81–220 *μ*g/dL) and those with platelets normal number (162.2 +/− 54.8 *μ*g/dL). No significant correlation was found between the platelet count and both the CIBDAI or CE pattern.

## 4. Discussion

In human medicine, hematological aspects of IBD and modifications of iron metabolism raise up great interest, because they are frequent complications that reduce the quality life of the patient, may worsen symptoms such as fatigability, weakness, nausea, and may be important factors involved in the pathophysiology of inflammatory response [1, 2, 13]. Iron deficiency mainly results from chronic blood loss in the intestine, but iron malabsorption due to inflammatory activity may also contribute [14]. Complex changes in iron metabolism, mediated by cytokines such as IL-1, IL-6, IFN-*γ*, and TNF-*α*, occur also in chronic inflammatory disease, with reduced release of iron from macrophages to transferrin. The classical biochemical picture includes both low serum iron and transferrin saturation level, and elevated serum ferritin concentrations [14].

In veterinary literature, mild anemia is reported in 12% of dogs with IBD [10], in agreement with our result where mild microcytic or normocytic anemia was found in 18% of dogs. Microcytic anemia is often related to iron deficiency due to chronic hemorrhage [15, 16], but, in our study, none of the anemic dogs had a positive fecal occult blood test or very low iron concentration or high TIBC concentration. These findings rule out an IDA. In dogs with occult fecal blood anemia, low iron or ferritin concentrations were not detected, whereas erythrocytosis and reticulocytosis observed in these cases support an erythroid compensatory response. On the other hand, a single positive occult fecal blood test does not provide information about the duration of intestinal hemorrhage. 

The MCV may be slightly decreased in association to chronic inflammatory disease [16]. In addition, the MCV appears frequently slightly below the reference range and Hct is frequently within the reference range or only a mild anemia is present [15]. In our study, microcytosis without anemia was the more frequent finding than anemia and, most of the dogs had an MCV at the lower end of the reference range. The MCV is not a sensitive parameter to detect iron bioavailability. Steinberg and Olver [17] reported that the evaluation of both reticulocyte Hgb content (CHr) and MCV (rMCV) would be more useful in evaluating iron-dependent erythropoiesis in dogs. Both low rMCV and CHr may occur in the earlier stages of iron depletion and may therefore be a sensitive markers of early iron unavailability.

The iron values at the lower end of the reference range, normal ferritin, normal or slightly low TIBC, and high CRP concentration support the diagnosis of anemia of chronic disease (ACD). The pathogenesis of ACD is mediated by cytokines produced during inflammation and resulted from iron sequestration, inducing reduction of erythrocyte half-life decline and erythropoietic response [16]. Serum ferritin and UIBC are acute-phase proteins that, in neoplastic and inflammatory diseases, may be increased and decreased, respectively. This feature likely decreases the sensitivity to detect iron deficiency. The use of others markers is convenient, such as serum transferrin receptor in human medicine, to characterize iron abnormalities [18–20]. Although Jergens and others [11] pointed out that CRP was a marker of disease severity in dogs with CE, in the present study CRP concentrations were not correlated with CIBDAI. On the other hand, this finding agrees with Allenspach and others [21], but more studies are needed to verify the role of CRP in canine CE. 

Although clinical improvement or complete recovery from symptoms was present in all dogs, and they were unrelated to CIBDAI or type of CE, erythrocyte parameters at posttreatment evaluation were not so improved as well. In addition, polychromasia, anisocytosis, and Howell Jolly bodies in RBCs observed in the blood smear, all together signs of bone marrow activation, were identified both in pre- and posttreatment evaluation, and CRP concentrations were not reduced significantly. 

It might be speculated that mild subclinical intestinal wall inflammation, not detectable by CIBDAI application, persists also in dogs recovered from symptoms. This hypothesis may be supported by several studies [21–23] where in cases of CE no significant changes were observed at posttreatment in the severity of histopathologic lesions in the face of an improvement of both clinical signs and endoscopical findings. 

Alternatively, considering that typical hematologic changes do not occur until late in severe iron abnormality [18], it is possible that anemia, microcytosis, and thrombocytosis needed more than the six months time of our study to improve. The significant increase of iron, UIBC, and TIBC serum concentrations revealed an improvement of iron metabolism with therapy. The observed modifications of iron and UIBC do not increase significantly the transferrin saturation post therapy, because it derives from the ratio iron/TIBC. Harvey and others [24] reported that steroid therapy may result in increased serum iron concentrations in dogs, but we did not observe any differences among SRD group versus FRD and ARD groups. 

In the present study, 31.8% of dogs showed thrombocytosis, whereas in a previous investigation Craven and others [10] observed it only in 7% of dogs. Thrombocytosis can be observed in several disorders including bone marrow neoplastic condition, even if rarely reported, infectious and inflammatory disorders, several neoplasia, acute bleeding or hemolysis, and iron deficiency [7]. Thrombopoietic growth factors including IL-6, TNF-*α*, and thrombopoietin have been implicated as causes of reactive thrombocytosis [7, 25].

In man, high platelet count and a low MPV are well-recognized markers of active IBD activity and probably represent a nonspecific response to inflammation [26], but iron abnormalities can also be involved, although the mechanism causing reactive thrombocytosis in iron deficiency anemia is currently not well understood [7]. In our small study, no significant differences were found in serum iron concentration in dogs with or without thrombocytosis, but more research is needed to unveil the mechanism responsible for the increase of platelets in dogs with CE. In human medicine, high plasma thrombopoietin concentrations were found in patients with ulcerative colitis and Crohn's disease [27], and both the procoagulant state and microvascular thrombosis may play a role in the pathogenesis of IBD [28]. 

In human medicine, a low MPV is associated with active IBD [27, 29], but in the present study microthrombocytosis was not revealed. In our study, no posttreatment significant reduction of platelet counts was observed and persistent mild intestinal wall inflammation might be the cause as suggested for the erythrocyte parameters. Finally, no significant correlations in platelets counts and MPV among CIBDAI versus FRD, ARD, and SRD were noted.

## 5. Conclusions

The significant modifications of iron metabolism in CE could encourage the evaluation of iron and UIBC serum concentrations, as nonspecific markers of inflammation. The use of more sensitive and specific markers of iron disorders, such as transferrin soluble receptors in human medicine [20], could be valuable in the future. The findings of this survey could stimulate a detailed approach to RBC and PLT features using more sophisticated laser cell counter to evaluate iron-dependent erythropoiesis in dogs.

## Figures and Tables

**Table 1:** Erythrocyte and platelet modifications pre- and posttreatment.

Test (units)	Reference Range	Pretreatment	Posttreatment	*P*
Hct (%)	37–55	44.4 ± 6.5	45.4 ± 6.0	.636
Hb (g/dL)	12–18.5	15.8 ± 2.2	16 ± 1.9	.801
MCV (fL)	60–76	63.0 ± 2.9	64.2 ± 2.8	.180
Reticulocytes (×10^9^/L)	<60.0	39.4 ± 18.8	37.5 ± 13.9	.736
PLT (×10^9^/L)	150–400	356.2 ± 135.7	324.6 ± 101.9	.390
MPV (fL)	7.9–12	10.2 ± 1.2	10.3 ± 1.0	.947

Hct, hematocrit; Hb, hemoglobin; MCV, mean cell volume; PLT, platelets; MPV, mean platelet volume.

**Table 2:** Serum iron profile and CRP modification pre- and posttreatment.

Test (units)	Reference Range	Pretreatment	Posttreatment	*P*
Iron (*μ*g/dL)	81–220	129.2 ± 34.8	166.0 ± 64.8	.030
UIBC (*μ*g/dL)	150–300	208.1 ± 48.9	258.5 ± 80.5	.010
TIBC (*μ*g/dL)	270–460	337.3 ± 64.7	424.5 ± 105.9	.001
Transferrin saturation (%)	25–52	38.5 ± 8.5	39.2 ± 9.9	.820
Ferritin (ng/mL)	60–190	116.9 ± 45.5	118.1 ± 48.9	.931
CRP (mg/dL)	0.0–0.15	0.31 ± 0.02	0.26 ± 0.39	.703

UIBC, unsaturated iron binding capacity; TIBC, total iron binding capacity; CRP, C-reactive protein.
